# Meat Quality Parameters, Sensory Properties and Consumer Acceptance of Chicken Meat from Dual-Purpose Crossbreeds Fed with Regional Faba Beans

**DOI:** 10.3390/foods11081074

**Published:** 2022-04-07

**Authors:** Cynthia I. Escobedo del Bosque, Stephanie Grahl, Tanja Nolte, Daniel Mörlein

**Affiliations:** 1 Department of Agricultural Economics and Rural Development, University of Goettingen, 37073 Goettingen, Germany; 2 Department of Animal Sciences, University of Goettingen, 37075 Goettingen, Germany; stephanie.grahl@agr.uni-goettingen.de (S.G.); tanja.nolte@uni-goettingen.de (T.N.); daniel.moerlein@uni-goettingen.de (D.M.); 3 Center for Integrated Breeding Research, University of Goettingen, 37075 Goettingen, Germany

**Keywords:** alternative protein source, Kollbecksmoor, preference, slow-growing, *Vicia faba*

## Abstract

Consumers’ concerns regarding the ethical and environmental practices of the current poultry production system have led to the search for an alternative production method. This study evaluated samples of three dual-purpose chicken crossbreeds: Vorwerkhuhn × Bresse Gauloise (VBG), Vorwerkhuhn × White Rock (VWR), and Bresse Gauloise × White Rock (BWR), fed with two variants of faba beans (vicin/convicin-rich and -poor: VC+ and VC−, respectively) and soybeans to examine whether the FB-based diets affected the meat quality of the crossbreeds. pH, color, water holding capacity, tenderness, nucleotide content and proximal composition were analyzed instrumentally, whereas sensory properties were identified by a trained panel and product acceptance was evaluated by frequent chicken consumers. Results showed that from instrumental measurements, the yellowness of the samples was affected by the type of feedstuff, whereas most other parameters were affected by the crossbreed, particularly color and nucleotide content. Sensory attributes, specifically, overall chicken aroma as well as firmness and crumbliness, were affected by an interaction of the feedstuff and crossbreed. Consumer preference did not show significant differences between samples. Overall, a faba-bean-based diet appeared to be a suitable alternative to a soybean-based diet on the crossbreeds VBG, VWR, and BWR when assessing the overall quality and taste of chicken breasts.

## 1. Introduction

Poultry meat production and consumption has grown worldwide for several years [1]. Germany has been a part of this trend, i.e., poultry consumption has increased by 4.12 kg per capita in the last ten years [2]. This growth has mainly been attributed to the general perception that this type of meat is healthier than red meat [3,4]. In the past few decades, specialized chicken breeds have been selected to achieve a higher performance at a comparably low cost [5]; therefore, separating production into fattening (meat-type) and laying (egg-type) lines. In the fattening line, both sexes are used for meat production, whereas in the laying line, only hens are used for egg production. Male layers do not produce enough meat (when compared with broilers); thus, they are deemed as “not profitable” and are therefore culled shortly after hatching [6]. In Germany, around 45 million male chicks from laying breeds are killed due to non-profitability each year [7]. This practice has raised ethical concerns among consumers in European countries, including Germany [8,9,10]. Stakeholders’ attention to this issue has led to the development of alternatives to this practice, including the use of dual-purpose breeds (DPBs). DPBs are breeds where females produce eggs and males produce meat [11,12]. However, DPBs lay fewer eggs and produce less meat than specialized breeds, even if they are kept for a longer period of time. This difference in performance makes it challenging to compete with the specialized breeds because DPBs lead to increased costs related to feed and housing [13] which implies higher product prices. Nonetheless, the general impression that such “non-conventional” production methods produce tastier, healthier, more animal-friendly and ethically justifiable alternatives could lead to consumers being willing to pay higher prices for such products [6,9,14]. Additionally, this topic is of particular relevance to the poultry industry, because in Germany, a law to prohibit the killing of day-old chicks took effect from the beginning of 2022 [7].

The selection of specialized chicken breeds has also led to a loss of genetic diversity in poultry breeding. The conservation of traditional breeds contributes to the preservation of genetic resources [15,16]. Nowadays, there are commercial (e.g., Lohmann Dual (Lohmann Breeders GmbH, Cuxhaven, Germany)) and traditional DPBs (e.g., Vorwerkhuhn, Bresse Gauloise) being used in Germany. However, in order to increase traditional DPBs’ laying and/or fattening performances, crossbreeding these traditional breeds with high-performing commercial breeds has been practiced to produce a DPB with a higher laying performance [17]. For instance, crossing the traditional breed Vorwerkhuhn (VH) with White Rock (WR) produces a DPB which has a higher laying performance than VH [18].

With the increased demand for chicken meat, the demand for protein-rich feedstuff is higher than what the European Union can produce; therefore, it needs to be imported from other countries [19,20]. Soybeans are largely used as a protein source in poultry diet formulations [21]. Nonetheless, soybean production is associated with deforestation, particularly in South America where, by 2016, 9% of forest loss was converted to soybean cultivation area [22]. Additionally, the dependency on its import is causing instability to local agriculture due to the price volatility of soybeans on the global market [23]. An alternative to soybeans as a protein source for feedstuff formulation is the use of other protein crops such as peas and beans. These crops are grown locally and therefore provide the agricultural sector with an opportunity to stop depending on soybean imports [23]. Faba beans (*Vicia faba* L.) are one of the most widely cultivated legumes [24]; their global production grew from 4.59 million tons in 2015 to 5.43 million tons in 2019 [25]. They provide the environmental benefits of improving soil fertility by fixating biological nitrogen which results in increased soil biological activity [23,26]. Faba beans are also highly nutritious due to their rich contents of K, Ca, Mg, and Fe [27,28], lysine [24,27], and protein (~30%) [29]. However, research has shown [21,30] that faba beans (FBs) also contain antinutritional factors, named vicin and convicin (together, VC), which limit their use in poultry diets. Additionally, results in the literature are inconsistent regarding the effect of FBs as a protein source in poultry diets, as it appears to depend on the genotype of the animal and on antinutritional characteristics of FBs [29], whose VC content depends on the specific cultivar [24]. Regarding laying hens, Laudadio et al. [31] and Dänner et al. [32] found that FB did not affect laying performance or egg quality when included in the diet of the hens; however, Koivunen et al. [33] found that FB decreased the egg weight when added to the diets of laying hens. In a more recent study [18], a VC-poor (0.022%) diet decreased the laying performance at the end of the laying period of the breed VH when compared with a soybean-based diet, while a VC-rich (0.138%) diet was intermediate; however, there was no difference in the other two breeds (Bresse Gauloise (BG) and WR) examined. Regarding fattening of the cockerels, Nolte et al. [34] found no significant effect on the animal growth and carcass performance of three different breeds (BG, VH, WR) fed with soybeans, VC-rich (0.14%), and VC-poor (0.02%) diets. An additional experiment by Escobedo del Bosque et al. [35] tested meat quality parameters (physicochemical and sensory) of breast samples from BG, VH, and WR fed with soybean- and FB-based feedstuff. It was found that, on occasion, small differences in meat quality could be attributed to the inclusion of FBs in the animals’ diet depending on the breed being examined. Generally, the presence of FBs improved meat quality.

The goal of this experiment was to examine whether the FB-based diets affect the meat quality of the crossbreeds of BG, VH, and WR. No adverse effects have been found in animal growth, carcass performance, and product quality (physicochemical and sensory analyses) of the pure breeds fed with two variants (VC-poor and VC-rich) of FB; therefore, we expect no adverse effect in the product quality of crossbreeds fed with FB.

## 2. Materials and Methods

This experiment was conducted in accordance with the European Union directive on the protection of animals used for scientific purposes (Directive 2010/63/EU) and was approved by the Lower Saxony State Office for Consumer Protection and Food Safety (LAVES; ref. 33.9-42502-04-17/2622). Additionally, all human participants (sensory assessors and consumers) gave written informed consent to take part in the study before it started. These studies were conducted in accordance with the Declaration of Helsinki, and the protocol was approved by the Ethics Committee of the University of Goettingen, Germany.

### 2.1. Animal Management and Sampling

In this study, the following crossbreeds were researched: VBG (Vorwerkhuhn males × Bresse Gauloise females), VWR (Vorwerkhuhn males × White Rock females), and BWR (Bresse Gauloise males × White Rock females). The chicks were hatched and reared for the first three weeks of life at the Institute of Animal Welfare and Animal Husbandry of the Friedrich-Loeffler-Institut in Celle, Germany, in indoor pens using a commercial starter diet. At 21 days, 120 chicks of each crossbreed were transported to the Department of Animal Sciences at the University of Goettingen (Goettingen, Germany), where 40 chicks of each crossbreed (10 per pen) were randomly assigned to one of three feed groups, generating a total of 9 different experimental groups (3 crossbreeds × 3 feed groups) with four replicates each. The animals were reared in an indoor-floor system, where each pen measure 2 × 1.5 m^.^ and was covered with wood shavings. The temperature was lowered from 22 °C to 20 °C and the photoperiod was 16 h.

All crossbreeds were subject to three different diets (Table 1) starting at day 21. The control diet (C) was based on soybean meal, whereas the other two diets were based on faba beans with different VC contents: one diet provided a high (0.136%) VC content (VC+), whereas the other consisted of a low (0.016%) VC content (VC−). Table 1 shows the ingredient composition of each diet. Feed and water were given ad libitum.

The animals were reared for 13, 14, and 15 weeks for VBG, BWR, and VWR, respectively, in order to reach a target live weight of approximately 2100 g. Details regarding the slaughter of the birds, as well as results related to growth and carcass performance, are reported in [34].

Half of the animals in each group (3 crossbreds × 3 feeds) were used for sensory analysis, and the other half were used to analyze physicochemical meat quality traits.

**Figure 1:**
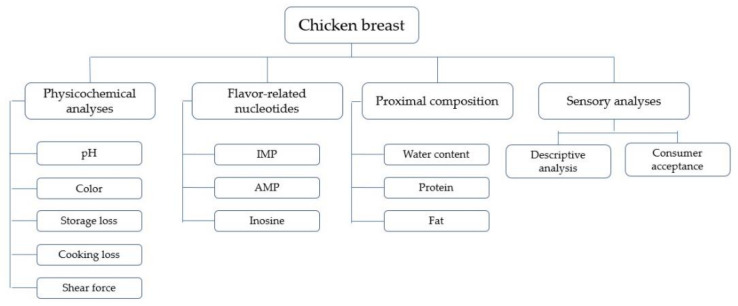
Scheme of the sample analyses.

Ten samples of each group were used to conduct the following analyses: pH, color, storage loss, cooking loss, instrumental tenderness (shear force), content of flavor-related nucleotides (i.e., inosine-5′-monophosphate (IMP), adenosine-5′-monophosphate (AMP) and inosine), and meat composition parameters (i.e., protein, intramuscular fat and water content). Figure 1 presents a scheme of the sample analyses. All experiments, except for proximate composition and consumer evaluation, were realized at the University of Goettingen.

### 2.2. Physicochemical Analysis

All breast samples were stored between 24 and 72 h postmortem (p.m.) in modified atmosphere (80% O_2_/20% CO_2_) packaging using a polypropylene (PP) tray with absorbent liners and heat-sealed with an oriented OPET/PP film (<3 cm^3^/m^2^/24 h bar O_2_ transmission rate; <12 cm^3^/m^2^/24 h bar CO_2_ transmission rate) using a vacuum packaging machine (TS 100, KOMET Maschinenfabrik GmbH, Plochingen, Germany) and stored at 4 °C without illumination.

The pH values were measured by inserting a pH electrode and a thermometer (Portamess 911, Knick Elektronische Messgeräte GmbH & Co. KG, Berlin, Germany) into the breast at 20 min p.m., 24 h p.m. and 72 h p.m. [36,37,38]. The instrument was calibrated with standard buffers for pH 4 and pH 7 (Carl Roth GmbH + Co. KG, Karlsruhe, Germany) at room temperature every time a new crossbreed was analyzed. The color of the samples was measured using CIELAB (L*a*b*; lightness, redness, and yellowness, respectively) coordinates by using a colorimeter (CR-600d, Konica Minolta, Tokyo, Japan), which was calibrated before each session with a white tile from the manufacturer, over non-overlapping areas [36,37,38]. The aperture size was 8 mm, and the illuminant D65 and standard observer angle was 10°. Color was recorded at 24 (breast with and without skin) and 72 h p.m. (breast without skin). Next, water holding capacity was measured as storage and cooking loss. Storage loss was measured by weighing the breast at 24 h p.m. and then at 72 h p.m., the percentage difference in weight was considered storage loss. Next, these samples were stored at −20 °C for 8 weeks p.m. until further analyses were conducted. Similar storage conditions have been previously tested in turkey and chicken breast meat [39,40] and no significant differences were found between fresh and frozen/thawed samples when testing cooking loss and shear force. Before further testing, for cooking loss and shear force analyses, samples were thawed overnight at 4 °C and re-packaged for immersion in a hot water (80 °C) bath (1092, GFL Gesellschaft für Labortechnik mbH, Burgwedel, Germany) for 50 min until reaching a core temperature of 76 °C. This temperature was measured by inserting a thermometer (926, Testo SE & Co. KGaA, Lenzkirch, Germany) into the sample. Once the samples reached room temperature, they were weighed; cooking loss was measured by weighting the samples prior to cooking and after cooking, and the percentage difference in weight was the cooking loss. After weighting, aluminum foil was used to wrap and store the samples overnight at 4 °C. The following day, once samples reached room temperature, shear force was measured according to Xiong et al. [41] with the following modifications: a TA.XTplus Texture Analyzer (Stable Micro Systems, Surrey, UK) equipped with a 5 kg load cell and a Meullenet-Owens Razor Shear Blade (MORS-Blade) [36,37]. The conditions for the test were: pretest speed 2 mm/s, test speed 10 mm/s, trigger type 10 g. Each sample was sheared four times perpendicular to the muscle fiber orientation, with a 1.5 cm distance from each cut. Shear force is reported as the peak shear force (N) needed to completely shear through the sample.

### 2.3. Flavor-Related Nucleotides

Samples of raw meat were collected at 24 h p.m., frozen with liquid nitrogen, and stored at −72 °C to later measure flavor-related nucleotide content. IMP, AMP, and inosine contents were determined six months p.m. by adapting Morzel and Van De Vis’ method [42]. Two hundred milligrams of minced samples were homogenized (Schuett-homgenplus homogenizer, Schuett-biotec GmbH, Goettingen, Germany) with 1 mL of 5% (*w*/*v*) trichloroacetic acid (TCA) (aq) (Carl Roth GmbH + Co. KG, Karlsruhe, Germany) for 1 min at 1600 rpm (Pico & Fresco 17/21 centrifuge, ThermoElectron LED GmbH, Osterode, Germany) and then chilled on ice for 15 min. The liquid extract was centrifuged at 4 °C for 5 min at 12,000× *g*. The supernatant (200 µL) was diluted 1:4 (*v*/*v*) with 5 % (*w*/*v*) TCA (aq) at pH 7.0. Extracts were kept at −20 °C before being injected into the high-performance liquid chromatography (HPLC) system (VWR Hitachi Chromaster, VWR International GmbH, Hannover, Germany), which was equipped with a 5260 pump, a 5260 autosampler (injection volume: 10 µL), and a 5410 UV detector operating at 260 nm. A LiChroCART Lichrosphere 100 RP-8 (250 × 4.6 mm, 5 µm) (Merck KGaA, Darmstadt, Germany) column was maintained at 30 °C in a 5310 column oven. The mobile phase consisted of 100 mM KH_2_PO_4_ (aq) (Carl Roth GmbH + Co. KG, Karlsruhe, Germany), 1.44 mM TBAHS (aq) (Sigma-Aldrich, Merck KGaA, Darmstadt, Germany), and 0.5% methanol (aq, pH 7.0) (VWR International GmbH, Hannover, Germany). The quantification was performed by an external calibration method and the identification of the analytes was performed by a comparison of retention times from the reference standards (Sigma-Aldrich, Merck KGaA, Darmstadt, Germany). All analyses were performed in duplicate.

### 2.4. Proximate Composition

To measure the contents of protein, intramuscular fat and water, 10 samples of breast meat from each group were removed at 72 h p.m., then homogenized 6 times at 1000 rpm for 30 sec in a grinder (Grindomix GM 200, Retsch GmbH, Haan, Germany), then vacuum packed in high-density polyethylene (HDPE) and stored at −20 °C. Nine months after slaughter, samples were thawed for 24 h and placed in a Petri dish where the concentrations of these parameters were obtained by near-infrared transmission spectroscopy using a Foss FoodScan™ (FOSS A/S, Hillerød, Denmark), following Anderson’s method [43].

### 2.5. Sensory Evaluation

All samples used for sensory analyses were stored at 24 h p.m. in MAP packaging, in the storage conditions stated in Section 2.2. Next, at 72 h p.m., they were vacuum-packed in polyamide/polyethylene bags and frozen at −20 °C until needed. Samples were then thawed overnight at 4 °C before use. Samples were cooked following the method to measure cooking loss (Section 2.2), then cut in 1 cm^2^ pieces and served on warm plates marked with a three-digit code

The sensory laboratory at the University of Goettingen (compliant with ISO 8589) was used to carry out all panel sessions. A trained panel of 9 assessors (7 female and 2 male) who had experience in creating a sensory profile of meat were trained and selected following international standard ISO8586. Before starting training and evaluation, all panelists signed a written consent form to participate.

Nine different products (three crossbreeds in three of each feed groups) were evaluated. All attributes that described the samples best were defined by the assessors, creating a list with 16 attributes on which they were further trained. Table A1 (Appendix A) presents a list with the attributes, definitions, and scales used to assess these samples. Product evaluation took place in five sessions, as each sample was evaluated three different times. In each session, panelists evaluated six samples in a sequential monadic manner. Four groups with a random order of samples were created, and each group was then assigned to two or three assessors. Between each sample, panelists used drinking water, unsalted crackers and cucumber for palate neutralization. A scale of 0 (no perception) to 100 (strong perception) was used to evaluate the samples. The data were collected using EyeQuestion (Version 4.8.7, EyeQuestion, Elst, The Netherlands).

### 2.6. Consumer Study

Consumers’ overall liking of samples was estimated using a nine-point hedonic scale [44] from “I do not like it at all” (1) to “I like it very much” (9). Then, consumers were presented a list of attributes (Table S1, Supplementary Material) from which they had to check all that apply (CATA) to describe the sample. The same procedure was repeated for consecutive samples. Between each sample, consumers neutralized their senses by drinking water; additionally, unsalted crackers were available for neutralization.

All consumer testing took place in a commercial sensory laboratory at ISI GmbH (Rosdorf, Germany). All samples used for the hedonic testing were cooked and served the same way as for the descriptive analysis (see Section 2.5). At the beginning of the test, consumers were informed that they would taste six chicken breast samples without any condiments. The reason for using no condiments was to avoid masking the mere flavor of meat, which was one of the points of interest in this study.

Due to a limited amount of material (meat) available, and in order to achieve a higher number of consumer responses, a balanced incomplete block design (BIBD) was generated where each consumer tasted six out of nine samples. The order of samples within each session was randomized to avoid first-order effect. The design divided samples into 3 groups (A, B, and C), where each group consisted of 6 samples (2 crossbreeds × 3 feed groups). Each crossbreed was evaluated with its 3 feed groups in order to avoid losing the interaction of crossbreed and feed. Table 2 outlines the design used for hedonic testing in more detail. Each session consisted of nine to twelve participants, who provided written informed consent prior to participation. Upon registration for the study, consumers were screened for their meat consumption, and only those that consumed chicken meat at least every two weeks were invited to participate in the test.

A total of 95 consumers (43 female and 52 male, aged 18 to 64 years) participated in the evaluation. Samples in group A were evaluated by 30 participants, samples in group B by 31 participants, and samples in group C by 34 participants. Therefore, each group was evaluated by at least 60 participants, which was the minimum number of participants required for consumer testing according to norm DIN 10974.

### 2.7. Statistical Analysis

Physicochemical parameters were analyzed using SPSS (IBM Corporation, New York, NY, USA) statistical software. Mean values were calculated, and significance effects were compared among all crossbreeds with a one-way ANOVA using Tukey’s multiple comparison statistical test at a 95% confidence level (α = 0.05). Sensory data was analyzed with the linear mixed model (LMM) procedure from SPSS. In the model, “product” was defined as a fixed effect, and “assessor”, “replicate”, and “replicate × assessor” were set as random effects. Within the model a Bonferroni statistical test at a 95% confidence level (α = 0.05) was used. Consumer overall liking (OL) was also analyzed using the LMM procedure; however, in the model, “crossbreed”, “feedstuff”, and “crossbreed × feedstuff” were defined as fixed effects, whereas “assessor” was set as a random effect. Within the model, a Bonferroni statistical test at a 95% confidence level (α = 0.05) was used. Due to the nature of the design (i.e., BIBD), CATA data had missing values for each sample (2.56% of total data), and were therefore imputed by calculating the median value using the multiple imputation procedure in SPSS. The data were then analyzed in XLSTAT-Sensory (Addinsoft, Paris, France), where Cochran’s *Q*-test with Sheskin means comparison tests was used to identify differences between the samples. Additionally, a partial least squares regression (PLSR) analysis was performed with standardized (1/SD) sensory data (x variables) and consumer data (y variables) using The Unscrambler X (Camo Analytics, Oslo, Norway) to identify relevant sensory attributes that drive the overall liking of consumers.

## 3. Results

The following section presents the results of physicochemical analyses, flavor-related nucleotides, proximate composition as well as sensory results of descriptive and affective testing.

### 3.1. Physicochemical Results

Table 3 shows the results for each physicochemical parameter for each group in detail. Few differences were found between the pH of the samples. pH at 24 h p.m. was mostly affected by the interaction between the feedstuff and the crossbreed, whereas the differences in pH of samples at 20 min p.m. and 72 h p.m. were attributed to a crossbreed effect. pH 20 min p.m. was lower in VBG than in VWR and BWR, whereas pH 72 h p.m. was lower in VBG than VWR. Color showed the most notable distinction between all samples, where the intensity of the yellowness (b*) of samples without skin measured at 72 h p.m. varied mainly due to an interaction effect of the crossbreed and feedstuff. Additionally, both measurements at 24 h p.m. (with and without skin) showed a difference in yellowness based on the feedstuff, where C is yellower than VC−. In contrast, most variations of all other color measurements between samples were due to a crossbreed effect; for instance, the a* and b* of BWR samples with skin differed from those of VBG and VWR with skin. As for the water holding capacity, there were no differences found between samples when measuring storage or cooking loss. Similarly, when measuring shear force, there were no significant differences found between samples.

### 3.2. Flavor-Related Nucleotides and Proximate Composition

Table 4 shows the results for nucleotide content and proximate composition for each sample in detail. The analysis and quantification of flavor-associated nucleotides showed a higher content of IMP, followed by inosine and AMP. Results showed a significant crossbreed effect in the content of these nucleotides, particularly in inosine content where VBG significantly differed from VWR and BWR, and in AMP content, where it was significantly lower in BWR than VBG and VWR. Table A2 (Appendix B) shows the methodological results of the nucleotide quantification. Regarding meat composition parameters, the water content did not differ between the samples. Protein content was affected by a crossbreed effect and a higher protein content was observed in BWR when compared with VBG. A difference in fat content was found between VC+ and VC− samples of VBG crossbreeds, and also between VC+ samples of VBG and VWR crossbreeds.

### 3.3. Sensory Results

Table 5 presents the results of the sensory evaluation (descriptive analysis) in detail. In breast samples, the fibrous appearance was affected by a crossbreed effect, whereas aroma was mostly affected by the crossbreed and feedstuff interaction. The attributes of overall aroma and chicken aroma presented significant differences between VBG and VWR. However, these differences were found between different feed groups: in VBG, C had a lower overall aroma than VC− and VC+. Similarly, chicken aroma in VBG × VC+ were different than other VBG and VWR samples. Regarding the flavor of the samples, differences were found in sourness due to a feedstuff effect, whereas the overall flavor intensity was affected by the interaction between crossbreed and feedstuff. Significant differences were found in the firmness of samples, where for the BWR crossbreed, C was firmer than the VC+ and VC− variants, as well as VBG × C, VBG × VC+ and VWR × VC− samples. These differences show an effect of the interaction between crossbreed and feedstuff. Similarly, crumbliness showed significant differences between crossbreeds and feedstuff, specifically VWR × C was significantly less crumbly than VWR × VC+ and BWR × C.

Participants appeared to prefer samples with C or VC+ feedstuff, because these obtained the highest percentages of mentions in “I like it very much” (highest score). In contrast, the most disliked samples were those with a VC+ diet and BWR breed. As observed in Table 6, the highest number of participants for each sample rated the meat between points 6 and 7, on the 9-point scale. Although there seemed to be a preference for VWR and BWR × C, when assessing the means for overall consumer liking, there were no significant differences between samples. However, a slight feedstuff effect (ɑ < 0.1) was present.

The results of Cochran’s Q test for each CATA attribute are presented in Table S1 in the Supplementary Material. Cochran’s Q test showed that the calculated *p*-value was lower than the significance level (α = 0.05) in the firmness of samples VBG × C and VWR × VC+, where the latter had a firmer texture. This difference was also noticed by the sensory panel. However, it did not seem to have an effect in the overall liking (OL) of the samples, as these do not statistically differ.

**Figure 2:**
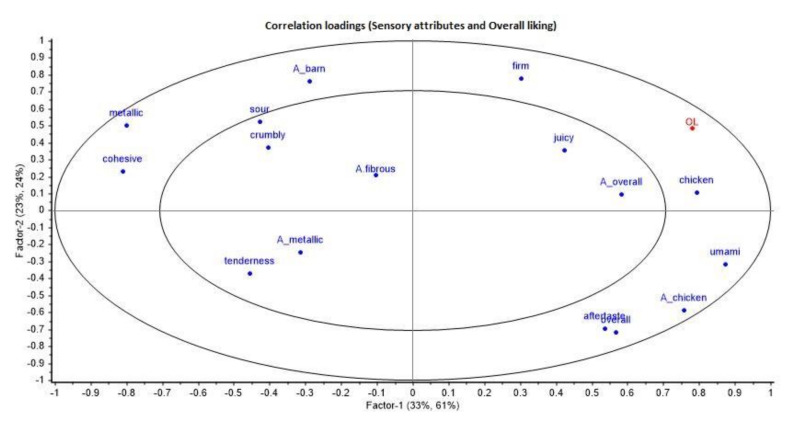
Correlation loadings for panel and consumer data.

The relationship between consumer preference and sensory characteristics was evaluated using a partial least squares (PLS) regression. The aim of the PLSR was to identify the most relevant chicken sensory attributes that influence the overall liking of the product. The results of the regression model are presented in Figure 2.

The correlation coefficient (r = 0.609) was investigated to see how well the model fitted the data. The model explained 56% of the sensory data (x) and 85% of the consumer overall liking (y). These results showed that chicken flavor, umami flavor, firmness, juiciness, chicken aroma, and overall aroma were positively associated with the OL of the samples. On the other hand, cohesiveness, tenderness, metallic flavor and aroma were negatively associated with the OL of the evaluated samples.

## 4. Discussion

As shown in [34], the breast yields of these crossbreeds are very similar, and no significant difference was found when comparing the different diets. Although yield performance is an important factor to consider when assessing the acceptability of a breed or feedstuff, there are also other defining parameters to consider. Hence, this study showed that using faba beans as a protein source is an acceptable alternative to soybeans, based on evaluating the physicochemical and organoleptic parameters of these samples.

Regarding the values of pH at 24 h p.m., no significant differences between the samples were observed. The values obtained in this study were slightly lower than in other studies with dual-purpose cockerels [36,45], which can be attributed to slaughter age, genetic factors, and slaughtering conditions. Moreover, in [35], similar pH values were found 20 min p.m. and slightly higher values in pH 24 h p.m. in the parent breeds (BG, VH, and WR) fed with the same diets, whereas [46] found similar values in BG and broilers at 12 weeks. Similarly, other studies that have tested soybean-based versus faba-bean-based diets in poultry have also reported no differences in the pH values of breast muscles [30,47]. Additionally, pH values of all samples at 20 min and 24 h p.m. do not indicate signs of pale, soft, and exudative (PSE) or dark, firm, and dry (DFD) incidence [48,49], which are unfavorable for the further processing of meat. Aside from pH, color is an important meat quality trait since it is usually considered by consumers to infer the quality of the product at the point of sale [3,50,51]. Similar values in the lightness of breasts from parent breeds [35] were observed; however, differences in redness and yellowness at 24 h p.m. were found. This difference in color could be attributed to genetics, because it is a factor that influences poultry skin and meat color [50,52]. Other studies also found large variations in breast color between different breeds, including dual-purpose breeds [36,53,54] and, consistent with the results of the present study, De Marchi et al. [55] and Almasi et al. [56] observed yellower skin and meat in indigenous breeds and slow-growing genotypes.

Characteristics such as WHC and instrumental tenderness are also of importance for meat quality. The values of WHC and instrumental tenderness of these crossbreeds were similar to those of the parent breeds [35]. However, in this study, we did not find any significant differences regarding these characteristics based on crossbreed or feedstuff.

The contents of AMP, IMP, and inosine are also of importance because these nucleotides, particularly IMP, are strongly associated with the increase in umami taste which is related to meat flavor intensity [57]. No differences were found between samples in the concentration of the abovementioned nucleotides. Similar to previous research [35,58,59,60], IMP was found in the highest concentration compared with the other nucleotides. A relevant IMP content was only found in fresh samples because AMP breaks down rapidly after slaughter [61]. Finally, the chemical composition of the samples was affected by the crossbreed and the interaction between crossbreed and feedstuff. The average moisture content of the samples was between 72% and 73%, similar to previous research with other genotypes [45,62]; however, it slightly differed (1–2%) from that of BG, a parent breed, in Muth et al.’s study [46]. The fat content in the samples was significantly influenced by the interaction effect of the crossbreed and the feedstuff. Additionally, similar results were obtained by Baéza et al. [63], where 12-week-old chickens (male and female from different genotypes) obtained values of intramuscular fat from 0.8% to 1.2%. Finally, when comparing the protein content in the breast muscles it became evident that there was a strong crossbreed effect, where BWR had the highest protein content, followed by VWR and VBG.

Similar to physicochemical characteristics, organoleptic properties are also strongly affected by genotype, feedstuff, and age [14,64,65,66]. Therefore, studies have usually focused on evaluating the organoleptic properties of meat from chickens of different breeds [35,36,67,68,69], fed with different diets [35,38,65], or reared in different production systems [14,65,66,70].

In this study, aroma differences were only present in crossbreeds of a Vorwerkhuhn parent, which is the only breed that showed differences in aroma attributes in a previous study [35]. The differences in the attributes of overall aroma intensity and chicken aroma are associated with an interaction effect of crossbreed and feedstuff. In overall flavor, the difference in VWR between soybean-based and faba-bean-based diets was also reflected in the fat content of the samples; a higher amount of intramuscular fat heavily influences meat flavor [71]. The texture attributes of firmness and crumbliness showed a significant interaction effect (crossbreed and feedstuff), particularly in firmness. Nonetheless, these results were not confirmed by the instrumental shear force test, where samples did not exhibit any significant differences. However, this might be due to the different testing temperatures of samples (instrumental shear force was at room temperature whereas sensory tests were in warm conditions), as consumers also indicated similar significant differences in the firmness of some samples via their CATA ratings.

Many odor, flavor, and texture attributes are present in different products; however, some attributes are particularly relevant for the overall liking of products. In the case of chicken meat, taste has been shown to have the greatest influence on the OL, followed by tenderness and juiciness, whereas the effects of aroma and color are less significant [72]. Similarly, Sow et al. [73] found that the attributes of juicy, oily, sweet, hard, mouth persistent, and yellow color were highly correlated with consumer preference, whereas tenderness, although positively correlated with OL, was not considered a relevant sensory driver of preference. Similar to these previous studies, our results also show the influence of flavor, texture and aroma attributes, in that order, in the correlation to OL. In this study, the flavor and aroma attributes which showed a positive correlation to OL were chicken aroma and flavor, as well as umami flavor, whereas metallic taste and aroma showed a negative correlation to OL; similar results were obtained by Horsted et al. [66]. This was also reflected in the frequencies of the top three boxes (T3B) of consumer acceptance, where VBG × VC−, VWR × C, VWR × VC+ and BWR × C also showed a higher intensity (in the sensory evaluation) of positively correlated attributes to OL, such as overall aroma and firmness. In this study, tenderness was negatively correlated to the OL of the samples, although it is usually positively correlated to the OL of meat products [74,75], including poultry [66,72,73]. This might be due to the type of production system, i.e., not a commercial breed and reared for a longer period of time, as Horsted et al. [66] also observed that niche production systems scored lower in tenderness when compared with standard products. Nevertheless, the negative association of tenderness to OL in these samples could be compensated by the positive association of firmness and juiciness to OL. The results of this study also indicated heterogeneous consumer liking, as a high variability between samples was observed (average standard deviation = 2) with respect to overall liking.

Based on the distribution of the hedonic assessment, a preference for C and VC+ feedstuff was observed. Although the frequency of T3B in BWR × VC+ was one of the highest, its value in the bottom three boxes (B3B) was the lowest. In most cases, the frequencies in the T3B were less than 50%, whereas those of the B3B were over 10%, even reaching up to 20% for BWR × VC+. This suggested that the samples were not particularly liked; however, this may have been due to the lack of seasoning in the samples. The use of spices and herbs has been shown to positively influence the OL of meat products, particularly of those with a low fat content [76,77], such as chicken breast.

Results of animal performance are of great importance for the breeding of dual-purpose chickens, because this helps determine their suitability as an alternative to current practices. To date, research shows that the use of faba beans does not affect growth and fattening performance; moreover, the BG breed and its crossbreeds were more suitable for meat production [34]. In our previous research with the parent breeds (BG, VH, and WR), the use of faba beans did not affect the quality parameters (instrumental and sensory) of the meat. Although differences between the breeds were found (e.g., BG was more tender, VH had a higher content of flavor-related nucleotides), it was still unclear whether these were due to the breed itself or to the 6-week age difference of the breeds [35]. However, in this study, most differences in physicochemical parameters were due to a crossbreed effect; because these differences were positively associated with quality for different breeds in different parameters (e.g., higher IMP content—associated with a more intense chicken flavor which is correlated to OL—in VWR when compared with BWR), based on these quality parameters, it is difficult to select a specific crossbreed that can be recommended for rearing dual-purpose breeds.

The results obtained are of relevance to the poultry industry because dual-purpose breeds are a viable alternative to the culling of day-old male chicks. However, they only apply to these specific crossbreeds fed with these particular feedstuffs. The production of these breeds (DPBs) also provides small-scale farmers an opportunity to target consumers that demand a more ethical production method, and who often indicate to be willing to pay higher prices for animal-friendly practices [6,9,14] and regional products [78,79,80] when these production systems do not compromise product quality.

## 5. Conclusions

The results of this study enabled us to quantify different meat quality parameters as well as identify sensory characteristics and consumer preferences for VBG, VWR, and BWR fed with vicin/convicin (VC)-rich and VC-poor faba-bean-based diets and a soybean-based diet. The analyses of meat quality parameters showed that the different crossbreeds, rather than feedstuff, were responsible for the slight differences in specific parameters. The sensory analysis showed that differences in attributes were mostly attributed to the interaction between the crossbreed and feedstuff, particularly in texture and aroma. Based on their sensory attributes, the samples were not clearly distinguished between each other by the panelists. Consumer evaluation showed that all samples were equally accepted by consumers; however, a VC-rich diet seemed to be better accepted than a VC-poor diet. Based on this study and on previous research, we conclude that a diet based on faba beans did not differ from a soybean-based diet when assessing the overall quality of chicken breasts; therefore, it can be used to substitute this protein source. The many differences between crossbreeds in different physicochemical parameters make it hard to recommend the rearing of a specific DPB; however, the lack of significant differences in sensory evaluation, including consumer acceptance showed that each of these crossbreeds can be used as DPBs without compromising hedonic quality.

## Figures and Tables

**Table 1:** Ingredient composition of each experimental diet.

	Control	Vicin+	Vicin−
Ingredients (%)			
Wheat	30.0	8.0	8.0
Corn	36.0	25.2	25.2
Soybean meal	24.4	-	-
Blue sweet lupines, cv. Boruta	-	28.6	28.6
Peas, cv. Astronaute	-	10.5	10.5
Faba beans, cv. Fuego	-	20.2	-
Faba beans, cv. Tiffany	-	-	20.2
Grass meal	5.6	0.1	0.1
Soybean oil	0.2	2.7	2.7
Dicalcium phosphate	1.3	2.2	2.2
Calcium carbonate	1.0	0.7	0.7
Salt (NaCl)	0.3	0.4	0.4
DL-Methionine	0.2	0.4	0.4
Vilomix Broiler premix 77047 ^1^	1.0	1.0	1.0
Chemical analyses			
Dry matter (%)	89.7	90.1	90.2
Ash (g/kg DM)	67.3	64.3	67.0
Crude protein (g/kg DM)	213.0	213.1	214.3
Crude fat (g/kg DM)	33.5	67.0	67.3
Crude fiber (g/kg DM)	45.2	72.0	74.0
Methionine (%)	0.46	0.50	0.49
Cysteine (%)	0.29	0.29	0.30
Lysine (%)	0.90	1.08	1.09

^1^ Vitamin–mineral premix provided per kilogram of diet: Fe, 32 mg; Cu, 12 mg; Zn, 80 mg; Mn, 100 mg; Se, 0.4 mg; I, 1.6 mg; Co, 0.64 mg; retinol, 3.6 mg; cholecalciferol, 0.088 mg; tocopherol, 40 mg; menadione, 4.5 mg; thiamine, 2.5 mg; riboflavin, 8 mg; pyridoxine, 6 mg; cobalamin, 32 µg; nicotinic acid, 45 mg; pantothenic acid, 15 mg; folic acid, 1.2 mg; biotin, 50 µg; choline chloride, 550 mg. Source: Adapted from [34].

**Table 2:** Balanced incomplete block design for breast samples tested with consumers (Product 1: VBG × C, Product 2: VBG × VC+, Product 3: VBG × VC−, Product 4: VWR × C, Product 5: VWR × VC+, Product 6: VWR × VC−, Product 7: BWR × C, Product 8: BWR × VC+, Product 9: BWR × VC−).

Group *	Session	Products		
A	1	1	2	3
		4	5	6
B	2	4	5	6
		7	8	9
C	3	1	2	3
		7	8	9
C	4	1	2	3
		7	8	9
A	5	1	2	3
		4	5	6
B	6	4	5	6
		7	8	9
B	7	4	5	6
		7	8	9
C	8	1	2	3
		7	8	9
A	9	1	2	3
		4	5	6

* Group A: Products 1–6, Group B: Products 4–9, Group C: Products 1–3 and 7–9. The order of samples within each session was randomized.

**Table 3:** Means, standard deviations and crossbreed, feedstuff and interaction effect for physicochemical parameters of each sample.

Parameter	Means and Standard Deviations									Effect					
	VBG			VWR			BWR			Crossbreed		Feedstuff		Crossbreed × Feedstuff	
	C	VC+	VC−	C	VC+	VC−	C	VC+	VC−	F	*p*	F	*p*	F	*p*
pH															
pH _20_	6.1 ± 0.20	6 ± 0.18	6 ± 0.19	6.2 ± 0.18	6.2 ± 0.19	6.1 ± 0.18	6.1 ^1^ ± 0.27	6.1 ± 0.14	6.3 ± 0.19	5.51	**	0.22	n.s.	1.91	n.s.
pH _24_	5.5 ± 0.08	5.6 ± 0.07	5.7 ± 0.10	5.6 ± 0.09	5.7 ± 0.25	5.6 ± 0.07	5.6 ± 0.13	5.6 ± 0.14	5.6 ± 0.11	0.03	n.s.	1.72	n.s.	2.74	*
pH _72_	5.5 ± 0.09	5.6 ± 0.07	5.6 ± 0.10	5.6 ± 0.09	5.8 ± 0.25	5.6 ± 0.05	5.6 ± 0.08	5.6 ± 0.09	5.6 ± 0.12	3.99	*	3.08	#	1.85	n.s.
Color with skin															
L* _24_	65.8 ± 3.71	63 ± 4.35	60.5 ± 5.49	64.9 ± 3.79	62.6 ± 4.49	64.4 ± 4.14	62.7 ± 5.85	61.4 ± 4.25	60.9 ± 2.55	2.16	n.s.	2.77	#	0.96	n.s.
a* _24_	2.5 ± 0.52	1.9 ± 1.11	2 ± 1.09	1.3 ± 0.90	2 ± 1.14	1.9 ± 0.99	0.7 ± 0.61	1.1 ± 0.90	0.6 ± 0.73	16.46	***	0.28	n.s.	1.54	n.s.
b* _24_	16.7 ± 2.73	15.1 ± 3.13	13.8 ± 2.07	13.9 ± 1.97	14.4 ± 3.31	14 ± 1.99	13.7 ± 3.18	12.4 ± 2.61	10.9 ± 3.05	8.48	***	3.53	*	0.966	n.s.
Color without skin															
L* _24_	64.2 ± 4.72	61.3 ± 3.96	61.0 ± 4.81	59.4 ± 3.19	59.8 ± 4.93	61.3 ± 3.71	59.7 ± 4.43	58.7 ± 3.08	58.9 ± 3.47	4.40	*	0.61	n.s.	1.00	n.s.
a* _24_	−0.1 ± 0.40	−0.1 ± 0.65	−0.1 ± 0.66	0.8 ± 0.82	0.5 ± 0.73	0.3 ± 0.51	−0.2 ± 0.49	−0.3 ± 0.35	0.1 ± 0.53	14.92	***	0.42	n.s.	1.47	n.s.
b* _24_	9.5 ± 1.43	10 ± 1.54	7.8 ± 2.65	11.4 ± 1.92	10 ± 1.18	9.5 ± 1.35	10.3 ± 1.62	9.8 ± 1.48	10.2 ± 1.93	4.09	*	3.69	*	2.00	n.s.
L* _72_	64.7 ± 3.64	62.4 ± 2.94	61.8 ± 4.03	59.6 ± 2.19	59 ± 3.99	60 ± 2.76	59.2 ± 3.72	57.5 ± 2.53	58.7 ± 2.75	15.59	***	1.93	n.s.	0.70	n.s.
a* _72_	0.6 ± 0.54	0.7 ± 0.55	0.9 ± 0.79	1.2 ± 0.64	1.2 ± 0.63	0.9 ± 0.36	1.0 ± 0.65	1.1 ± 0.50	1.3 ± 0.44	3.80	n.s.	0.08	n.s.	0.85	n.s.
b* _72_	9.4 ± 1.88	10 ± 1.31	8.2 ± 1.85	9.6 ± 1.34	8.6 ± 1.60	8.7 ± 1.00	8.1 ± 1.82	8.4 ± 1.27	9.6 ± 1.69	0.79	n.s.	0.12	n.s.	3.78	**
Water holding capacity															
Storage loss (%)	2.2 ^1^ ± 0.55	2 ^1^ ± 0.24	1.8 ^1^ ± 0.13	2.1 ± 0.25	4.8 ^1^ ± 5.86	1.9 ± 0.32	1.8 ^1^ ± 0.13	1.7 ± 0.28	1.8 ± 0.38	2.92	#	2.14	n.s.	2.30	#
Cooking loss (%)	22.3 ± 1.54	21.5 ± 1.01	22 ± 1.76	19.6 ± 7.41	20.3 ± 1.55	21.3 ± 1.18	20.8 ± 1.79	20.9 ± 1.14	21.4 ± 0.94	2.28	n.s.	0.54	n.s.	0.37	n.s.
Instrumental tenderness															
Shear force (N)	4.8 ± 1.12	4.8 ± 0.74	4.7 ± 1.05	5.3 ± 0.83	5.1 ± 0.84	4.7 ± 0.81	4.3 ± 1.30	4.6 ± 1.22	4.5 ± 0.71	2.32	n.s.	0.44	n.s.	0.46	n.s.

VBG = Vorwerkhuhn × Bresse Gauloise, VWR = Vorwerkhuhn × White Rock, BWR = Bresse Gauloise × White Rock, C = control, VC+ = high in vicin, VC− = low in vicin. ^1^ *n* = 9 due to missing measurements at time of observation. *, **, ***: *p* < 0.05, 0.01, 0.001, respectively; #: *p* < 0.10; n.s.: not significant.

**Table 4:** Means, standard deviations and crossbreed, feedstuff and interaction effect for flavor-related nucleotides and proximal composition of samples.

Parameters	Means and Standard Deviations									Effect					
	VBG			VWR			BWR			Crossbreed		Feedstuff		Crossbreed × Feedstuff	
	C	VC+	VC−	C	VC+	VC−	C	VC+	VC−	F	*p*	F	*p*	F	*p*
Nucleotides (mg/100 g)															
IMP	303 ± 31	264 ± 40	279 ± 42	320 ± 18	272 ± 42	295 ± 26	249 ± 34	273 ± 51	259 ± 36	3.60	*	1.20	n.s.	1.40	n.s.
AMP	9 ± 3	10 ± 4	12 ± 5	10 ± 2	9 ± 3	9 ± 2	7 ± 2	6 ± 1	6 ± 1	6.79	**	0.22	n.s.	1.00	n.s.
Inosine	29 ± 8	20 ± 2	24 ± 8	16 ± 4	15 ± 4	19 ± 5	17 ± 3	17 ± 2	20 ± 5	9.61	***	2.10	n.s.	1.81	n.s.
Chemical composition (%)															
Protein	23.8 ^1^ ± 1.2	23.6 ± 1.31	23.4 ± 1.41	24.9 ± 0.36	24.7 ± 0.77	24.7 ± 0.65	25.2 ± 0.43	25.2 ± 0.38	25.2 ± 0.33	27.40	***	0.47	n.s.	0.17	n.s.
Fat	0.9 ^1,abc^ ±1.33	1.1 ^a^ ± 1.49	0.7 ^bc^ ± 1.6	1.1 ^ac^ ± 0.33	0.7 ^b^ ± 0.81	0.8 ^abc^ ± 0.66	1 ^abc^ ± 0.39	1 ^abc^ ± 0.34	1 ^abc^ ± 0.28	2.05	n.s.	2.60	#	4.79	**
Water	72.8 ^1^ ± 1.48	72.8 ± 1.67	72.7 ± 1.78	72.8 ± 0.38	72.5 ± 0.92	72.6 ± 0.76	73.1 ± 0.46	73 ± 0.39	72.7 ± 0.32	2.21	n.s.	1.02	n.s.	0.30	n.s.

VBG = Vorwerkhuhn × Bresse Gauloise, VWR = Vorwerkhuhn × White Rock, BWR = Bresse Gauloise × White Rock, C = control, VC+ = vicin-rich, VC− = vicin-poor. ^1^ *n* = 9 due to missing measurements at time of observation. ^a, b, c^ Values with differing superscript letters are statistically significantly different (*p* < 0.05). *, **, ***: *p* < 0.05, 0.01, 0.001, respectively; #: *p* < 0.10; n.s.: not significant.

**Table 5:** Means, standard deviations and crossbreed, feedstuff and interaction effect for sensory attributes.

Attributes	Means and Standard Deviations									Effect					
	VBG			VWR			BWR			Crossbreed		Feedstuff		Crossbreed × Feedstuff	
	C	VC+	VC−	C	VC+	VC−	C	VC+	VC−	F	*p*	F	*p*	F	*p*
Appearance															
Fibrousness	34.6	34.8	38.0	28.0	28.2	29.8	37.7	35.7	33.7	45.39	*	3.72	n.s.	0.05	n.s.
Aroma															
Barn	52.0	60.5	54.3	51.7	54.7	52.5	56.1	54.4	54.5	1.19	n.s.	1.90	n.s.	1.37	n.s.
Metallic	30.7	30.3	29.2	28.3	28.1	28.2	25.0	29.3	24.6	2.40	#	0.61	n.s.	0.51	n.s.
Chicken	21.5 ^ab^	14.9 ^b^	27.1 ^a^	26.9 ^a^	21.7 ^ab^	19.0 ^ab^	20.8 ^ab^	22.2 ^ab^	22.8 ^ab^	0.19	n.s.	1.65	n.s.	3.01	*
Overall	52.2 ^a^	55.0 ^ab^	56.7 ^b^	56.3 ^ab^	56.8 ^b^	53.0 ^ab^	55.1 ^ab^	55.5 ^ab^	55.4 ^ab^	0.30	n.s.	0.67	n.s.	2.46	*
Taste															
Sour	51.5	57.7	50.2	50.7	54.8	52.2	50.8	50.7	48.0	2.00	n.s.	3.21	*	0.82	n.s.
Chicken	34.4	33.3	35.0	40.1	30.3	31.4	36.6	34.2	31.6	0.011	n.s.	2.51	#	1.09	n.s.
Metallic	47.1	53.8	46.4	41.4	48.8	50.2	47.4	47.4	44.8	0.90	n.s.	2.55	#	1.85	n.s.
Umami	23.0	19.5	23.6	27.0	22.1	21.4	23.6	22.9	22.0	0.34	n.s.	1.53	n.s.	0.78	n.s.
Overall	42.9	38.4	45.6	46.0	44.3	40.0	39.0	42.9	42.3	0.55	n.s.	0.10	n.s.	2.69	*
Aftertaste	33.0	31.6	34.1	34.7	33.7	32.6	31.3	32.8	32.7	1.12	n.s.	0.12	n.s.	1.24	n.s.
Texture															
Firmness	43.7 ^a^	49.7 ^bc^	49.0 ^abc^	47.1 ^abc^	50.0 ^bc^	45.5 ^ab^	51.1 ^c^	45.5 ^ab^	45.5 ^ab^	0.00	n.s.	0.58	n.s.	3.61	**
Juiciness	34.8	35.5	33.6	36.6	31.5	33.9	36.6	33.8	36.1	0.51	n.s.	1.23	n.s.	0.74	n.s.
Cohesiveness	49.1	50.3	47.5	45.2	48.8	50.5	49.6	52.0	47.7	0.64	n.s.	1.63	n.s.	1.67	n.s.
Tenderness	54.2	51.3	49.6	51.4	51.0	54.1	51.5	53.1	55.1	0.47	n.s.	0.253	n.s.	1.39	n.s.
Crumbliness	48.9 ^ab^	49.3 ^ab^	50.6 ^ab^	45.9 ^a^	52.6 ^b^	51.3 ^ab^	52.5 ^b^	49.4 ^ab^	48.5 ^ab^	0.07	n.s.	0.47	n.s.	2.80	*

VBG = Vorwerkhuhn × Bresse Gauloise, VWR= Vorwerkhuhn × White Rock, BWR = Bresse Gauloise × White Rock, C = control, VC+ = vicin-rich, VC− = vicin-poor. Mixed model: fixed effect: crossbreed, feedstuff, crossbreed × feedstuff, random effects: assessor, replicate, replicate × assessor. ^a, b, c^ Values with differing superscript letters are statistically significantly different (*p* < 0.05). *, ** : *p* < 0.05, 0.01, respectively; #: *p* < 0.10; n.s.: not significant.

**Table 6:** Consumers’ acceptance of chicken meat.

Breed	VBG (*n* = 64)			VWR (*n* = 61)			BWR (*n* = 65)			Effect		
Diet	C	VC+	VC−	C	VC+	VC−	C	VC+	VC−	C	F	C × F
Hedonic scale (% of participants)												
I like it very much (9)	6.3	9.4	9.4	11.5	8.2	4.9	7.7	6.2	6.2			
(8)	12.5	14.1	18.8	18	18	16.4	21.5	13.8	10.8			
(7)	25	26.6	12.5	24.6	16.4	18	20	27.7	26.2			
(6)	26.6	14.1	25	18	21.3	21.3	20	15.4	16.9			
Neither like nor dislike (5)	4.7	10.9	6.3	8.2	6.6	6.6	12.3	7.7	6.2			
(4)	12.5	10.9	15.6	13.1	19.7	19.7	10.8	9.2	15.4			
(3)	3.1	10.9	6.3	6.6	4.9	8.2	4.6	6.2	10.8			
(2)	4.7	0	4.7	0	3.3	4.9	1.5	7.7	6.2			
I dislike it very much (1)	4.7	3.1	1.6	0	1.6	0	1.5	6.2	1.5			
Top 3 boxes (T3B)	43.8	50.0	40.6	54.1	42.6	39.3	49.2	47.7	43.1			
Bottom 3 boxes (B3B)	12.5	14.1	12.5	6.6	9.8	13.1	7.7	20.0	18.5			
Overall liking	5.9 ± 2.02	6.0 ± 2.01	6.0 ± 2.04	6.4 ± 1.75	6.0 ± 1.97	5.8 ± 1.91	6.2 ± 1.82	5.7 ± 2.26	5.6 ± 2.05	n.s.	#	n.s.

VBG = Vorwerkhuhn × Bresse Gauloise, VWR = Vorwerkhuhn × White Rock, BWR = Bresse Gauloise × White Rock, C = control, VC+ = vicin-rich, VC− = vicin-poor. Top 3 boxes: sum of the highest 3 responses in the scale; Bottom 3 boxes: sum of the lowest responses in the scale. #: *p* < 0.10; n.s.: not significant.

## Data Availability

The data presented in this study are available on request from the corresponding author.

## Supplementary Materials

The following supporting information can be downloaded at: https://www.mdpi.com/article/10.3390/foods11081074/s1, Table S1: Multiple pairwise comparisons between samples and Cochran’s Q test in attributes used in CATA.

## Appendix A

**Table A1:** Sensory attributes, definitions and scales used to evaluate samples.

Attribute	Definition ^1^	Scale
*Odor*		
Overall intensity	The sum of all perceptible odors.	Not perceptible—Very perceptible
Animal/barn	The intensity of smell of animal/stable.	Not perceptible—Very perceptible
Metallic	The intensity of smell of metal/blood.	Not perceptible—Very perceptible
Cooked chicken	The intensity of smell of cooked, unseasoned chicken, chicken soup.	Not perceptible—Very perceptible
*Appearance*		
Fibrousness	Degree of visible fibers on the cut side of the sample.	Not recognizable—Very recognizable
*Taste*		
Overall intensity	The sum of all perceptible flavors.	Not perceptible—Very perceptible
Sour	The intensity of sourness.	Not perceptible—Very perceptible
Umami	The intensity of umami taste.	Not perceptible—Very perceptible
Cooked chicken	The intensity of the taste of cooked, unseasoned chicken or chicken soup.	Not perceptible—Very perceptible
Metallic	The intensity of the taste of metal or blood.	Not perceptible—Very perceptible
Aftertaste	The intensity of the aftertaste.	Not perceptible—Very perceptible
*Texture*		
Firmness	Force required to bite through the piece with the incisors.	Soft—Firm
Juiciness	Amount of fluid released during the first three chews.	Not juicy—Very juicy
Cohesiveness	Cohesion of the sample during chewing.	Not cohesive—Very cohesive
Tenderness	Force required to chew the piece until it can be swallowed.	Not tender—Very tender
Crumbliness	Number of pieces formed before swallowing; how strongly the mass holds together or decays during chewing.	Not crumbly—Very crumbly

^1^ Definitions were suggested and accepted by the panelists.

## Appendix B

**Table A2:** Analytical performances of nucleotide quantification.

	RT (min)	LoD (µg/mL)	LoQ (µg/mL)	Intraday (CV%)	Inter-day (CV%)	Dynamic Linear Range (µg/mL)	Calibration Equation	R^2^
IMP	3.89 ± 0.07	0.17	0.51	2.28	9.32	0.5–1000	Y = 22325x − 37939	0.9997
Inosine	4.8 ± 0.1	0.31	0.95	5.00	9.47	0.5–500	Y = 60623x − 183766	0.9994
AMP	9.7 ± 0.1	1.17	3.56	5.00	9.47	5–1000	Y = 33235x + 346258	0.9941
