# Supplementary material for: Meat Quality Parameters, Sensory Properties and Consumer Acceptance of Chicken Meat from Dual-Purpose Crossbreeds Fed with Regional Faba Beans
Source: Foods. 2022 Apr 7;11(8):1074. doi: 10.3390/foods11081074 (PMC9028872; doi:10.3390/foods11081074)
Supplement: Supplementary file 1 [file foods-11-01074-s001.zip › foods-1636680-supplementary.pdf]

Meat Quality Parameters, Sensory Properties and Consumer Acceptance of Chicken Meat from Dual-Purpose Crossbreeds Fed With Regional Faba Beans

Cynthia I. Escobedo del Bosque, Stephanie Grahl, Tanja Nolte, Daniel Mörlein

Supplementary Materials

Table S1. Multiple pairwise comparisons between samples and Cochran's Q test in attributes used in CATA.

	Vorwerkhuhn x Bresse Gauloise			Vorwerkhuhn x White Rock			Bresse Gauloise x White Rock			Cochran's Q
Attribute	C	VC+	VC-	C	VC+	VC-	C	VC+	VC-	p-value
Intense aroma	0.092	0.031	0.185	0.154	0.077	0.108	0.154	0.185	0.185	0.090
Weak aroma	0.308	0.292	0.323	0.400	0.462	0.354	0.354	0.338	0.308	0.571
Metallic flavor	0.154	0.108	0.077	0.077	0.092	0.092	0.108	0.077	0.077	0.876
Intense chicken flavor	0.292	0.246	0.246	0.277	0.262	0.169	0.277	0.308	0.277	0.838
Weak chicken flavor	0.277	0.385	0.323	0.323	0.338	0.446	0.415	0.462	0.369	0.416
Sour flavor	0.092	0.138	0.077	0.046	0.092	0.077	0.108	0.123	0.108	0.804
Aftertaste	0.215	0.169	0.108	0.138	0.154	0.185	0.246	0.108	0.185	0.436
Bland	0.200	0.169	0.185	0.185	0.215	0.108	0.169	0.215	0.154	0.853
Barn flavor	0.015	0.046	0.015	0.015	0.031	0.046	0.077	0.108	0.046	0.170
Rancid flavor	0.015	0.031	0.015	0.015	0.015	0.031	0.015	0.062	0	0.514
Bitter flavor	0.123	0.015	0.062	0.062	0	0.062	0.031	0.077	0.077	0.111
Firm	0.200^{a}	0.292^{ab}	0.215^{ab}	0.385^{ab}	0.462^{b}	0.277^{ab}	0.246^{ab}	0.323^{ab}	0.262^{ab}	0.027
Soft	0.277	0.231	0.323	0.231	0.185	0.262	0.354	0.308	0.308	0.495
Tough	0.169	0.200	0.138	0.138	0.154	0.123	0.108	0.138	0.108	0.883
Tender	0.308	0.231	0.354	0.354	0.215	0.246	0.323	0.262	0.338	0.500
Juicy	0.246	0.308	0.323	0.292	0.231	0.308	0.323	0.215	0.277	0.836
Dry	0.431	0.385	0.323	0.323	0.462	0.369	0.323	0.277	0.415	0.395
Cohesive	0.154	0.138	0.169	0.123	0.108	0.169	0.077	0.138	0.108	0.808
Crumbly	0.062	0.092	0.138	0.123	0.185	0.138	0.169	0.185	0.092	0.354
Aromatic	0.169	0.169	0.185	0.215	0.154	0.138	0.246	0.246	0.231	0.721
Pale color	0.415	0.446	0.462	0.354	0.308	0.431	0.492	0.446	0.385	0.474
Intense color	0.062	0.031	0.062	0.062	0.046	0.015	0.031	0.077	0.046	0.805

Multiple comparisons using critical difference (Sheskin) procedure. C=control, VC+ = high in vicin, VC-= low in vicin.

^{a,b,c} Means followed by differing superscript letters are statistically significantly different (α=0.05).
